# Social support as a coping resource for psychosocial conditions in postpartum period: a systematic review and logic framework

**DOI:** 10.1186/s40359-024-01814-6

**Published:** 2024-05-28

**Authors:** Khadijeh Khademi, Mohammad Hossein Kaveh

**Affiliations:** 1 Student Research Committee, Department of Health Promotion, School of Health, Shiraz University of Medical Sciences, Shiraz, 71536-75541 Iran; 2 Research Center for Health Sciences, Department of Health Promotion, School of Health, Institute of Health, Shiraz University of Medical Sciences, Shiraz, Iran

**Keywords:** Social Support, Postpartum, Depression, Quality of Life, Mental Disorders

## Abstract

**Purpose:**

This review seeks to examine the current state of postpartum social support and psychosocial conditions among women around the world, as well as explore the relationship between these factors. Additionally, it aims to propose a logical framework for enhancing postpartum social support and psychosocial conditions in this population.

**Methods:**

Following the development of a search strategy, two databases, PubMed and Science Direct, were searched for studies published between January 2019 and May 2023. The search was conducted throughout the entire month of May 2023. The risk of bias in the included cross-sectional studies was assessed using the Newcastle–Ottawa Quality Assessment Scale, which was adapted for this specific study design. To determine if the main objective of the cross-sectional studies was to investigate the relationship between social support and postpartum psychosocial conditions, a review was conducted based on the AMSTAR checklist, PRISMA checklist and PRISMA flow diagram. Data extraction was performed with the consensus of two authors, and a narrative synthesis approach was chosen for data synthesis, following the guidelines provided by the Centre for Reviews and Dissemination (CRD).

**Results:**

Eleven cross-sectional studies were included in the final analysis. Our findings revealed that all reviewed studies provided evidence of a positive association between social support and healthy psychosocial conditions in postpartum period. However, due to the absence of standardized measurement indicators to identify and compare the outcomes of various studies, there was a need to develop a conceptual framework that could enhance our understanding of the postpartum psychosocial condition including anxiety, depression, unfavorable quality of life and social support status up to 24 month after child birth. This framework aimed to incorporate childbirth and motherhood as "stressful events," while considering social support as a crucial "coping resource." Furthermore, it acknowledged empowerment, help-seeking behavior, and peer support as important "coping actions," alongside implementing client-centered interventions. Lastly, it recognized postpartum mental health and optimal quality of life as significant "effects" of these factors.

**Conclusions:**

The proposed conceptual framework could define postpartum women’s health as “the ability to adapt and self-manage.”

## Introduction

Birth and motherhood are considered neuro-psycho-social events, and the first year postpartum, although a pleasant time for a family, is a crucial developmental life stage for women [1, 2]. During this transitional period, women face a dual challenge: Firstly, they must adapt to the changes in their physical appearance and the added expectations of new responsibilities. Secondly, they experience physical discomforts resulting from pregnancy and childbirth, potential marital discord, and negative social interactions such as conflict, insensitivity and interference, especially with their husbands [2–4]. These changes and problems can be challenging and far-reaching, resulting in undesirable psychosocial well-being or psychosocial problems [5–7].

Psychosocial well-being is a superordinate construct that includes emotional or psychological well-being, as well as social and collective well-being [5]. So, postpartum psychosocial problems are defined as anxiety, depression, other mental disorders, unfavorable quality of life and increasing the incidence of suicidal ideation and behavior [1, 5–7]. Postpartum depression (PPD) is a type of major depressive episode that occurs during pregnancy or within 4 weeks following delivery [8]. Another related illness is postpartum post-traumatic stress disorder (PP-PTSD), which is an anxiety disorder that can develop as a result of a difficult or traumatic birth experience. It's important to note that women may experience PP-PTSD even after a successful birth [9]. Both PPD and anxiety disorder are serious psychosocial problems that require proper diagnosis and treatment [8, 9]. Notably, postpartum psychosocial problems can impede successful adjustment to the maternal role, impact the entire family, and impose significant economic costs on the healthcare system [10–12].

However, current postpartum care, which is often standardized in terms of content and structure, is associated with low satisfaction rates among mothers [13]. Furthermore, most studies focusing on mothers’ health outcomes in the postpartum period have centered on measures such as breastfeeding rates, hospital admissions, and physical health indicators of mothers and infants [14–17]. While these outcomes are undoubtedly important, mothers have also indicated that maternal functioning and mental health are essential for health [14, 18, 19]. To aid in coping with the stress of motherhood and promote maternal well-being, effective social support can be instrumental in facilitating a successful transition to motherhood [20–22].

Theoretical frameworks suggest that social support, which refers to the availability of others to provide emotional, psychological, and material resources, can serve as a coping resource that impacts health [23]. In addition, social support is also recognized as a protective factor against stress, anxiety, depression, and other mental disorders that can negatively impact the quality of life [24–26]. Negative postpartum health outcomes highlight the critical need for increased support and resources [14].

Further investigation is necessary to deepen our understanding of the status of social support after childbirth and how it can contribute to maternal psychosocial well-being during the transition to motherhood [20]. Although psychosocial conditions are crucial, because of persistent mother’s mental disorders up to 24 months after childbirth, they are often not the primary focus of research [24, 25]. Mothers’ low satisfaction rates with standardized postpartum care also highlight the need for a client-centered approach [13]. Ultimately, by identifying evidence-based factors that affect women’s psychosocial conditions and the necessity for plans in this area, postpartum can be one of life’s most pleasant experiences, and the challenges confronting them can be an opportunity for personal growth and skill enhancement [11].

In fact, the majority of current studies on postpartum women primarily focus on physical issues such as postpartum bleeding or pain, as well as psychological problems like breastfeeding difficulties and PPD [15–17]. Unfortunately, other psycho-social conditions that arise after childbirth are often overlooked and receive little attention [14, 24, 25]. To enhance the psycho-social well-being of women following childbirth, it is crucial to gain a comprehensive understanding of these conditions and assess the factors that influence them [11, 18]. Among these factors, social support plays a significant role and warrants further investigation to determine its importance during the postpartum period [20, 26].

Based on the background above, the current review aims to achieve the following objectives: 1) to explore evidence for the status of women’s social support in postpartum period (up to 24 month after child birth), 2) to assess the evidence for the status of women’s psychosocial problems including anxiety, PPD and unfavorable quality of life up to 24 month after child birth, 3) to evaluate the evidence for associations between social support and psychosocial problems in postpartum period, and 4) to propose a logical framework for enhancing postpartum social support and psychosocial conditions.

## Materials and methods

The present study utilized a systematic review based on the Preferred Reporting Items for Systematic Reviews and Meta-Analysis (PRISMA) [27] checklist, We chose two databases based on recommendations from the PRISMA group paper, which suggests searching at least one database, and the Assessing the Methodological Quality of Systematic Reviews (AMSTAR) checklist, which recommends searching at least two databases [28, 29]. Our search included well-known databases PubMed and Science Direct, and we used the following search strategy: keywords for each search term (Table 1), Field “Title/Abstract,” free full-text (access to some scientific articles is limited for Iranian universities due to debt, not having a subscription, etc.). Our investigation covered a period of five years, from January 2019 to May 2023. This timeframe was chosen to ensure that our evidence syntheses were as up-to-date as possible when published, as this is often neglected by authors of overviews [30].

**Table 1:** Keywords for each search term

**Term 1** Social support		**Term 2** Psychosocial conditions		**Term 3** Post-partum
**OR** Support	AND	**OR** mental disorders	AND	**OR** postnatal
**OR** family Support		**OR** depression		**OR** maternal
**OR** Psychosocial Support		**OR** stress		**OR** after childbirth
		**OR** anxiety		
		**OR** psychological distress		
		**OR** psychological problems		
		**OR** mental health		
		**OR** quality of life		
		**OR** psychosocial well-being		

### Study criteria

The inclusion criteria for the studies consisted of four key factors: (1) the participation of postpartum mothers up to 24 months, as research has shown that mothers can continue to experience mental disorders for up to 24 months after giving birth. This extended timeframe highlights the need for ongoing support and care for postpartum mothers beyond the traditional 12-month postpartum period [25]; (2) the use of self-administered questionnaires or scales to assess social support; (3) publication in full-text English; and (4) a descriptive or cross-sectional designs. Since, according to the Oxford Center's table of Evidence-Based Medicine 2011 and Dehkordi AH et al. review study, a review of descriptive or cross-sectional studies is necessary to examine the status of a medical or health condition and its relationship with other conditions [31, 32].

Exclusion criteria included studies that were: (1) clinical/controlled trials, qualitative, longitudinal, or non-original articles; (2) solely for psychometric purposes; and (3) targeted specific groups of postpartum women.

**Fig. 1:**
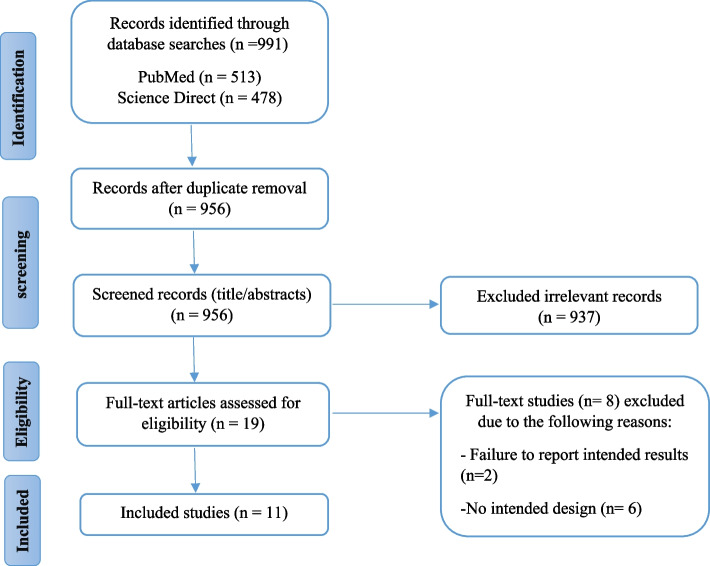
Study search and selection process

A total of 991 articles were initially identified in various databases and imported into EndNote X7 software [33], with duplicates removed. Out of the 956 articles, 19 papers were selected for inclusion after being assessed for relevance by two independent reviewers who achieved consensus on which studies to include [27]. Eight full-text studies were excluded due to failure to report intended results (*n* = 2) and no intended design (*n* = 6). Finally, 11 papers were reviewed. The study search and selection process are illustrated in Fig. 1.

### Risk of *bias* in included studies

**Fig. 2:**
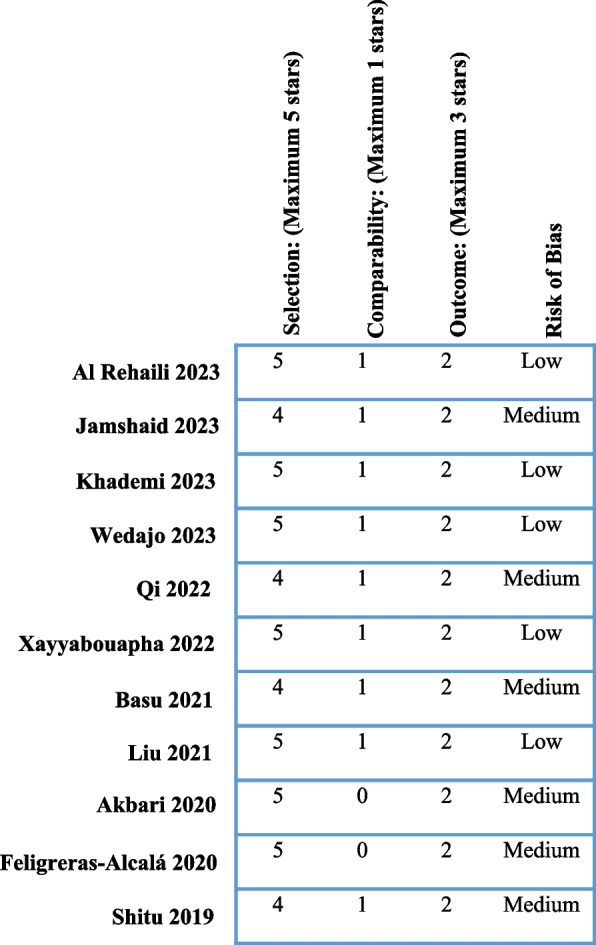
Summary of risk of bias; authors' assessment of the risk of bias for each study

The Newcastle–Ottawa Quality Assessment Scale (adapted for cross-sectional studies) [34] was used to determine the risk of bias. Five studies (45%) were determined to have a low risk of bias, 6 of them (55%) were a medium risk of bias, and no study (0%) was assessed as having a high risk of bias. Figure 2 presents the details of the risk-of-bias items separately for each article.

### Data synthesis

A meta-analysis is often not feasible due to the presence of significant heterogeneity in quantitative indices or measurement tools. In such cases, a narrative approach to synthesis may be more appropriate and effective [32, 35]. So, in present study a narrative and deductive approach to synthesis was selected, following the steps outlined in the Centre for Reviews and Dissemination (CRD). These steps included developing a preliminary synthesis of the results of included studies, exploring relationships in the data, and considering the robustness of the synthesis [36].

## Results

### Description of studies

Eleven cross-sectional studies were included in the final analysis, and the majority of studies (*N* = 8) took place in Asian nations. Only one study was conducted in Europe, while two were conducted in Africa. The maximum time after childbirth was 18 months. The characteristics of the studies are shown in Table 2.

**Table 2:** Characteristics of studies on the relationship between social support and psychosocial conditions

Authors, Ref	Country/ year	Aim of study	Number of participants	Age (Mean ± SD)	Time after child birth	Social support measurement	Psychosocial measurement	Statistical analysis—Result
Al Rehaili et al.[37]	Saudi Arabia 2023	To determine factors associated with postpartum quality of life (QOL)	252	31.3 ± 6.2	≤ 6 months	MSPSS^1^	WHOQOL-BREF^2^ EPDS^3^	Multiple linear regression—The significant other subscale of the MSPSS^1^ was significantly associated with higher QOL scores. In addition, postpartum depression (PPD) was significantly associated with lower QOL
Jamshaid et al.[38]	Pakistan 2023	To evaluate the association of PPD with insomnia, mental health, and physical health	320	-	2- 4 weeks	MSPSS^1^	EPDS^3^ WEMWBS^4^	Multiple linear & Hierarchal regression—Perceived social support has an important role in PPD. Additionally, it plays a significant moderating role in the relationship between PPD and mental health
Khademi et al.[39]	Iran 2023	To assess the impact of family social support (FSS) on postpartum quality of life (PQOL)	240	31.34 ± 5.52	1- 18 months	FSSQ^5^	PQOL^6^	Structural equation modeling (SEM)—FSS was associated with PQOL
Wedajo et al.[40]	Ethiopia 2023	To assess the prevalence of late postpartum depression and associated factors	479	28.82 ± 6.10	6 weeks- 12 months	MSSS^7^	EPDS^3^	Multi-variable logistic regression model—Low maternal social support were significantly associated factors with PPD
Qi et al.[41]	China 2022	To examine the relative contribution of marital satisfaction, perceived caring of the mother-in-law, and social support on postpartum depression and sleep quality simultaneously in a path model	817	-	6 weeks	SSRS^8^ PCMIL^9^	EPDS^3^	Path analysis—Enhancing social support might reduce postpartum depression
Xayyabouapha et al.[42]	Lao PDR 2022	To examine the prevalence and risk factors associated with postpartum depressive symptoms among women after delivery	521	27.5 ± 5.8	4- 24 weeks	MSPSS^1^	EPDS^3^	Multivariate logistic regression -Moderate social support was significantly associated with depressive symptoms
Basu et al.[43]	India 2021	To estimate the burden of postpartum depression and associated factors in women having an infant child	210	25.95 ± 3.74	< 1 year	MSPSS^1^	EPDS^3^	Binary logistic regression model—Women reporting low and medium levels of perceived social support had significantly higher odds of having postpartum depression
Liu et al.[8]	China 2021	To determine the prevalence of PPD and postpartum post-traumatic stress disorder (PP-PTSD), and to examine the relationships between a range of sociodemographic, pregnancy-related, and newborn-related variables, and PPD and PP-PTSD	1136	30.16 ± 3.91	6- 8 weeks	PSSS^10^	EPDS^3^ PPQ^11^	Multivariate logistic regression model—The presence of PP-PTSD was the strongest risk factor for PPD symptoms and vice versa. In addition, low social support is a risk factor for PP-PTSD and PPD
Akbari et al.[44]	Iran 2020	To investigate the relationship between spiritual well-being (SWB) and perceived social support with postpartum depression in new mothers	200	26.23 ± 5.59	4–8 weeks	MSPSS^1^	EPDS^3^ SWBS^12^	Logistic regression—Social support and SWB as a protective factor against postpartum depression
Feligreras-Alcalá et al.[45]	Spain 2020	To investigated the relationship between personal and family resources (i.e., social support) and depressive and anxiety symptoms in women during the puerperium	212	32.67 ± 4.58	6 weeks	Duke-UNC-11	EPDS^3^ STAI^13^	Multiple linear regression—Low perceived social support was proposed as a risk factor of anxiety and depression
Shitu et al.[46]	Ethiopia 2019	To show the prevalence and factors associated with PPD among postpartum mothers	596	30.57 ± 6.3	2 weeks- 12 months	MSSS^1^	EPDS^3^	Multivariate logistic regression model—Low social support was independent predictors of postpartum depression

^1^*MSPSS* Multidimensional Scale of Perceived Social Support

^2^*WHOQOL-BREF*: World Health Organization Quality of Life Assessment-BREF

^3^*EPDS* Edinburgh Postnatal Depression Scale

^4^*WEMWBS* Warwick–Edinburgh Mental Wellbeing Scale

^5^*FSSQ* Family Social Support Questionnaire

^6^*PQOL* Postpartum Quality of Life

^7^*MSSS* Maternity Social Support Scale

^8^*SSRS* Social Support Rating Scale

^9^*PCMIL* Perceived caring of Mother-in-law

^10^*PSSS* Perceived Social Support Scale

^11^*PPQ* Perinatal Post-Traumatic Stress Questionnaire

^12^*SWBS* Spiritual Well-Being Scale

^13^*STAI* State-Trait Anxiety Inventory

### Aim 1: Status of Postpartum Social support

Five studies used the Multidimensional Scale of Perceived Social Support (MSPSS) to assess social support. In comparison, two studies utilized the Maternity Social Support Scale (MSSS), and the remaining employed different questionnaires (see Tables 2 and 3).

**Table 3:** Status of postpartum social support and psychosocial conditions

Authors, Ref	Social support			Psychosocial conditions		
	**Tools (Total score)**	**Mean Score ± SD**	**Frequency**	**Tools (Total score)**	**Mean Score ± SD**	**Prevalence**
Al Rehaili et al.[37]	MSPSS^1^ (1- 7)	5.3 ± 1.2	-	WHOQOL-BREF^2^ (0- 100) EPDS^3^ (0- 30)	95.2 ± 16 9.9 ± 5.3	- -
Jamshaid et al.[38]	MSPSS^1^ (12- 84)	41.17 ± 5.24	-	WEMWBS^4^ (14- 70) EPDS^3^ (0- 30)	26.2 ± 4.85 22.56 ± 4.84	- -
Khademi et al.[39]	FSSQ^5^ (0- 100)	69.80 ± 11.19	-	PQOL^6^ (0- 100)	61.63 ± 9.59	-
Wedajo et al.[40]	MSSS^7^ (6- 30)	-	Low (score < 18): 15.97% Medium (score 18- 23): 23.63% High (score 24- 30): 60.40%	EPDS^3^ (0- 30)	-	*PPD* (score ≥ 13): 29.08%
Qi et al.[41]	SSRS^8^ (12- 66) PCMIL^9^ (1- 10)	38.42 ± 5.58 6.85 ± 2.88	-	EPDS^3^ (0- 30)	8.58 ± 5.49	*PPD* (score ≥ 10): 41.49% *PPD* (score ≥ 13): 23.13%
Xayyabouapha et al.[42]	MSPSS^1^ (1- 7)	-	Low (score < 3): 0% Medium (score 3- 5): 56.1% High (score > 5): 43.9%	EPDS^3^ (0- 30)	-	*PPD* (score ≥ 10): 21.3%
Basu et al.[43]	MSPSS^1^ (1- 7) Trichotomized score	-	Low (lowest scores): 27.2% Medium (middle scores): 33.3% High (high-scoring): 39.5%	EPDS^3^ (0- 30)	7.5 ± 6.1	*PPD* (score ≥ 10): 29%
Liu et al.[8]	PSSS^10^ (12- 84)	-	Low (score 12- 36): 1.9% Medium (score 37- 60): 31.4% High (score 61- 84): 66.6%	EPDS^3^ (0- 30) PPQ^11^ (0- 56)	9.54 ± 4.46 6.30 ± 6.37	*PPD* (score ≥ 13): 23.5% *Anxiety* (score ≥ 19): 6.1%
Akbari et al.[44]	MSPSS^1^ (12- 84)	-	*Reported as Low:* 73.3% *Reported as Medium*: 27.8% *Reported as High*:13.7%	EPDS^3^ (0- 30) SWBS^12^ (20- 120)	-	*PPD* (score ≥ 13): 22% *SWB in non-depressive:* Low (score 20- 40): 2.56% Medium (score 41- 99): 25% High (score 100- 120): 72.43%
Feligreras-Alcalá et al.[45]	Duke-UNC-11 (11- 55)	Median: 47.5	-	EPDS^3^ (0- 30) STAI^13^ (0- 60)	7.20 17.26 ± 10.64	*PPD* (score ≥ 10): 26.9% *Anxiety* (score > Q3^14^): 27.8%
Shitu et al.[46]	MSSS^1^ (6- 30)	-	Low (score < 18): 34.6% Medium (score 18- 23): 53% High (score 24- 30): 12.4%	EPDS^3^ (0- 30)	-	*PPD* (score ≥ 8): 23.7%

^1^*MSPSS* Multidimensional Scale of Perceived Social Support

^2^*WHOQOL-BREF* World Health Organization Quality of Life Assessment-BREF

^3^*EPDS* Edinburgh Postnatal Depression Scale

^4^*WEMWBS* Warwick–Edinburgh Mental Wellbeing Scale

^5^*FSSQ* Family Social Support Questionnaire

^6^*PQOL* Postpartum Quality of Life

^7^*MSSS* Maternity Social Support Scale

^8^*SSRS* Social Support Rating Scale

^9^*PCMIL* Perceived caring of Mother-in-law

^10^*PSSS* Perceived Social Support Scale

^11^*PPQ* Perinatal Post-Traumatic Stress Questionnaire

^12^*SWBS* Spiritual Well-Being Scale

^13^*STAI* State-Trait Anxiety Inventory

^14^*Q3*  ≥ 75th percentile

In two studies, the majority of women reported their postpartum social support level as medium (56.1% and 53%) [42, 46]. In three other studies, most women reported high-level social support (ranging from 39.5% to 66.6%) [8, 40, 43]. However, one study found that 73.3% of participants had low social support [44]. Additionally, in two studies, the authors interpreted the level of postpartum social support based on its mean score as moderate and high [37, 39]. It is important to note that the scoring for social support varied across these studies, as shown in Table 3.

### Aim 2: Status of postpartum psychosocial conditions

The Edinburgh Postnatal Depression Scale (EPDS) was utilized in all, but one of the studies [39]. The prevalence of Postpartum Depression (PPD) with a score of ≥ 13 was reported as 22%- 29.08% in four of the studies [8, 40, 41, 44]. Meanwhile, three studies reported the prevalence of PPD with a score of ≥ 10 as 21.3%- 41.49% [41–43, 45]. Furthermore, one study reported the prevalence of PPD with a score of ≥ 8.5 as 23.7% [46] (Table 3).

The prevalence of anxiety was assessed using the Perinatal Post-Traumatic Stress Questionnaire (PPQ) with a score of ≥ 19 and the State-Trait Anxiety Inventory (STAI) with a score of ≥ 75 percentile, reported rates of 6.1% and 27.80%, respectively [8, 45] (Table 3). The PPQ is a self-rating scale to identify women suffering from PTSD symptoms (i.e. re-experiencing, avoidance- numbing, and hyperarousal) at 1–18 months postpartum [8].

The level of mental health was assessed using the Warwick-Edinburgh Mental Wellbeing Scale (WEMWBS) in a study, with authors interpreting the mean score as low [38] (refer to Table 3 for details).

Only three studies explored the postpartum quality of life using the World Health Organization Quality of Life Assessment-BREF (WHOQOL-BREF), Spiritual Wellbeing Scale (SWBS), and Postpartum Quality of Life (PQOL). In a study, the level of PQOL was interpreted as medium based on its mean score [39]. Additionally, findings from a separate study showed that 72.43% of non-depressive women had a high score (100–120) on SWB [44] (Table 3).

### Aim 3: Association between postpartum social support and psychosocial conditions

Ten studies were identified that investigated the association between social support and postpartum depression (PPD), with results showing that social support was a statistically significant factor in the development of PPD [8, 37, 38, 40–46]. Only three studies, however, evaluated the link between women’s social support and mental health disorders such as anxiety and PTSD in postpartum period. These studies suggested that low levels of social support were a contributing risk factor for postpartum anxiety and other mental illnesses [8, 38, 45] (the statistical tests used are given in Table 2).

Moreover, three studies specifically examined the association between social support and quality of life in the postpartum period, where an association was confirmed [37, 39, 41, 44]. Furthermore, two of these studies found that this relationship was reciprocal [39, 44] (the statistical tests used are given in Table 2).

**Fig. 3:**
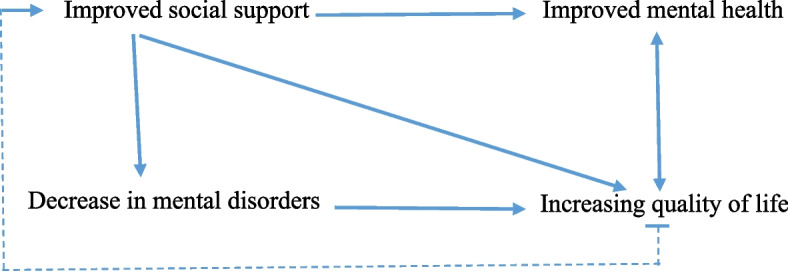
Relationship between social support and psychosocial conditions in postpartum period

Figure 3 summarizes the studies, analyses, and syntheses pertaining to the relationship between social support and psychosocial conditions in the postpartum period.

## Discussion

In this study, we systematically reviewed cross-sectional reports on the relationships between social support and psychosocial conditions in postpartum period. This review pursued four aims: 1) to explore evidence for social support status in postpartum period, 2) to assess the evidence for psychosocial conditions status in postpartum period, 3) to evaluate the evidence for associations between social support and psychosocial conditions in postpartum period, and 4) to propose a logic framework for improving postpartum social support and psychosocial conditions.

Regarding aim 1, our review found that the social support status of the majority of women in postpartum period was at a moderate to high level. There was a sufficient level of social support, as the majority of evidence came from Asia, where the appropriate level of support for postpartum women is associated with socio-cultural norms. For example, in Saudi culture, it is common for a mother to spend the puerperium period, especially the first forty days, at her mother's house to receive the necessary support until she recovers [37]. However, more research is needed to fully understand mothers’ social support in the postpartum period, as this is crucial in developing strategies to improve maternal health [47].

Concerning aim 2, our review found that the prevalence of PPD (EPDS score ≥ 13) was 22- 29.08%, but there was insufficient evidence to determine the prevalence of other mental disorders. Similar to our findings, the aggregate data meta-analyses (AGMAs) results of the systematic review studies revealed a prevalence of 17.22% to 27% for PPD [21, 48–51]. PPD is a major mental health issue that impacts about 1 in 8 women, as the transition to motherhood is not always smooth and can be overwhelming [52]. During this transitional period, women encounter numerous challenges including changes in their physical appearance, increased expectations of new responsibilities, physical discomforts from pregnancy and childbirth, and potential marital discord leading to psychosocial issues, particularly PPD [2–4]. Furthermore, PPD is a widespread social health concern that not only affects the mother and her newborn, but also has repercussions for other family members and various aspects of their lives [48, 49]. Consequently, there is a need to implement routine postpartum screening for maternal mental health to ensure early detection and treatment of PPD [53].

Regarding aim 3, our systematic review indicated that low social support is a significant predictor of self- reported PPD and anxiety symptoms. Furthermore, our findings demonstrated that social support and postpartum quality of life were mutually dependent. These results are consistent with previous studies, including review or meta-analysis studies with random- effects model, which have identified a lack of social support as a major risk factor for PPD [49–51, 54, 55]. Riem et al.’s review study also supports our results, as it found that low social support is associated with self- reported mental disorders symptoms, such as stress and anxiety, during postpartum [7]. Postpartum stress and anxiety are crucial to address as they have been linked to negative health behaviors in women, such as unhealthy diet, smoking relapse, and postpartum weight retention. Additionally, they are key factors associated with PPD, which can develop into self- reported major depression and pose significant risks to morbidity and mortality if left underdiagnosed [56]. It is important to recognize and address these issues in order to support the overall well-being of new mothers [52]. Social support is essential in reducing the risk of postpartum psychosocial problems by providing a protective effect. This includes feeling understood, accepted, and respected, which can help alleviate individual psychological pressure, inhibit negative emotions, and provide positive emotional experiences. Additionally, social support can aid in bonding and coping by improving self-evaluation, helping to form a positive self-image, and promoting self-esteem. Furthermore, social support can act as a buffer against the negative effects of stressors during the postpartum period [52, 56]. As such, providing adequate social and psychological support from family members, providing reassurance, conducting appropriate educational interventions, and regularly assessing the psychological state of women by healthcare providers can help them adapt to postpartum changes, improve their quality of life, and enhance their overall health [57].

### Aim 4: A proposed logic framework for improving postpartum social support and psychosocial conditions

**Fig. 4:**
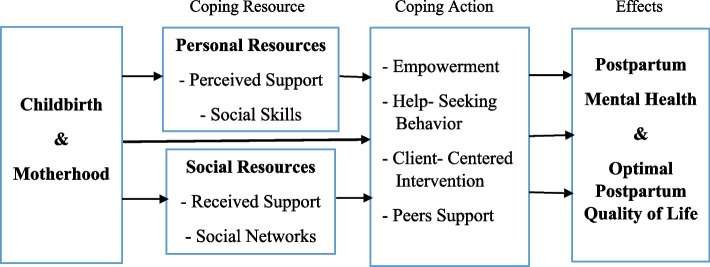
Proposed logic framework for improving postpartum social support and psychosocial conditions

Social support was protective against all postpartum psychosocial disorders; therefore, these findings provide a foundation for developing and tailoring interventions and strategies to improve mental health outcomes [58, 59]. The major components of our proposed logic framework are depicted in Fig. 4 and described below.

In the logic framework, stressful events are associated with childbirth and motherhood [1–4]. Social support is the coping resource in this framework [22]. According to this coping resource, during the coping action stage, researchers and healthcare providers should attempt to improve perceived social support by encouraging and empowering women to be actively involved in adapting to the physical and psychosocial changes occurring, acquiring the necessary knowledge, engaging in help-seeking behavior, and developing novel social roles throughout the postpartum period [18, 60–62]. In addition, health policymakers should be aware that flexible education-care-support planning is a promising tool for facilitating more client-centered care during the postpartum care period. In other words, it is crucial to transition from narrow-minded to comprehensive plans that integrate care, education, and support, as well as from uniformity to customization based on individual conditions, resources, and requirements. This shift entails prioritizing clients' personal needs and preferences over organizational constraints, which can help address challenges like escalating healthcare expenses and staffing deficiencies in care facilities. Also, it is consistent with the proposed positive definition of health, which shifts from total well-being to the “ability to adapt and self-manage.” As a result, health professionals encourage patients to participate in their own care processes more than ever before [13, 47].

Finally, the effects of this framework are optimal postpartum mental health and quality of life [58, 59].

The present systematic review is limited by its focus on English-language publications of the included studies. Additionally, a meta-analysis was not conducted due to shortcomings in quantitative indices and varying measuring tools. Nonetheless, this review provides a valuable and broad geographic overview of social support for postpartum psychosocial conditions. The novel contribution of this study is the development of a conceptual framework that offers insights into designing programs for postpartum mental health and quality of life. We recommend using standard protocols for designing, implementing, and evaluating interventions to facilitate comparison in systematic review and meta-analysis studies. Our proposed logic framework can be tested as a guiding framework in intervention design. We also recommend conducting a systematic review that focuses on specific types of action, such as empowerment. Client-tailored and risk-based maternity cares are two important strategies that empower postpartum women [63]. These interventions are designed to address the physical and psychosocial changes that women experience during this time, and they encourage women to actively participate in adapting to these changes [18]. By tailoring care to each individual's needs and addressing potential risks, women can feel more supported and empowered as they navigate the postpartum period.

## Conclusions

In conclusion, this systematic review indicates a positive association between social support and postpartum psychosocial conditions. Social support is viewed as a coping mechanism during the postpartum period, which can result in improved quality of life and mental health. This is achieved by empowering women, promoting help-seeking behaviors, and providing client-centered care. As such, the “ability to adapt and self-manage” defines postpartum women’s health.

## Data Availability

The datasets used and/or analyzed during the current study are available from the corresponding author upon reasonable request.
